# Validating a Shortened Depression Scale (10 Item CES-D) among HIV-Positive People in British Columbia, Canada

**DOI:** 10.1371/journal.pone.0040793

**Published:** 2012-07-19

**Authors:** Wendy Zhang, Nadia O’Brien, Jamie I. Forrest, Kate A. Salters, Thomas L. Patterson, Julio S. G. Montaner, Robert S. Hogg, Viviane D. Lima

**Affiliations:** 1 British Columbia Centre for Excellence in HIV/AIDS, St. Paul’s Hospital, Vancouver, British Columbia, Canada; 2 Department of Psychiatry, University of California San Diego, San Diego, California, United States of America; 3 Faculty of Medicine, University of British Columbia, Vancouver, British Columbia, Canada; 4 Faculty of Health Sciences, Simon Fraser University, Burnaby, British Columbia, Canada; University of Nebraska Medical Center, United States of America

## Abstract

**Objective:**

To establish the reliability and validity of a shortened (10-item) depression scale used among HIV-positive patients enrolled in the Drug Treatment Program in British Columbia, Canada.

**Methods:**

The 10-item CES-D (Center for Epidemiologic Studies Depression Scale) was examined among 563 participants who initiated antiretroviral therapy (ART) between August 1, 1996 and June 30, 2002. Internal consistency of the scale was measured by Cronbach’s alpha. Using the original CES-D 20 as primary criteria, comparisons were made using the Kappa statistic. Predictive accuracy of CES-D 10 was assessed by calculating sensitivity, specificity, positive predictive values and negative predictive values. Factor analysis was also performed to determine if the CES-D 10 contained the same factors of positive and negative affect found in the original development of the CES-D.

**Results:**

The correlation between the original and the shortened scale is very high (Spearman correlation coefficient  = 0.97 (P<0.001). Internal consistency reliability coefficients of the CES-D 10 were satisfactory (Cronbach α = 0.88). The CES-D 10 showed comparable accuracy to the original CES-D 20 in classifying participants with depressive symptoms (Kappa = 0.82, P<0.001). Sensitivity of CES-D 10 was 91%; specificity was 92%; and positive predictive value was 92%. Factor analysis demonstrates that CES-D 10 contains the same underlying factors of positive and negative affect found in the original development of the CES-D 20.

**Conclusion:**

The 10-item CES-D is a comparable tool to measure depressive symptoms among HIV-positive research participants.

## Introduction

Depression is a major problem among people living with HIV/AIDS [1], [2]. The Center for Epidemiologic Studies Depression Scale (CES-D) is one of the most common screening tests for identifying depressive symptoms in the general population [3]. The 20-item scale is a self-reported measure of an individual’s depressive feelings and behaviors in the past week. It is designed for studies ascertaining the relationship between depression and other variables. Major components of depressive symptomology are incorporated into the scale including, depressed mood, feelings of guilt and worthlessness, feelings of helplessness and hopelessness, psychomotor retardation, loss of appetite, and sleep disturbance [3]. The scale’s validity and reliability to detect both clinical and non-clinical depressive symptoms have been well established [3]. Moreover, the scale has been widely used in different cohort studies suggesting its suitability for a variety of sample populations and diverse contexts.

Despite the CES-D scale’s established reliability among general populations, when embedded in large-scale surveys, the 20-item measurement tool is lengthy and onerous. A few studies have shown that the CES-D could be reduced to 10-items with comparable reliability and validity to the original scale, though none with entire cohorts of HIV-positive individuals [4]–[7]. Previous studies have used various depression scales, such as the Hospital Anxiety and Depression Scale (HADS) [7], the Beck Depression Inventory (BDI) [8] and the Hamilton Rating Scale [9] to assess HIV-positive persons. While the BDI has been validated as a shortened scale to increase utilization, research has found that the questions in the BDI are depressing and too focused on the body [10], [11]. HIV is a condition that spans physical, mental, emotional, social and environmental spheres of health, thus the CES-D scale was selected for use in our research study. The validation of the 10-item CES-D scale may aid survey administration and reduce interviewee burden, especially for HIV patients, many of who are marginalized and face literacy limitations and have competing life demands.

Depression in HIV patients is often under-identified and under-treated which may become very harmful to HIV patients. A meta-analysis of ten studies investigating the relationship between HIV infection and depressive disorders found the frequency of events was nearly two times higher in people living with HIV compared to HIV-negative individuals [12]. Furthermore, studies have demonstrated that the presence of depressive symptoms in HIV-positive individuals is associated with earlier mortality and increased HIV-specific morbidity, despite access to HAART [1], [13]–[16]. Specifically, depressive symptoms have been found to be associated with key clinical markers of disease progression such as reduced CD4 counts [14], increased activated CD8 lymphocyte counts [17] and reduced virologic suppression for people accessing therapy [18].

The purpose of this study is to validate the shortened 10-item CES-D scale among a cohort of HIV-positive individuals.

## Methods

### Ethics Statement

Ethical approval was granted by the Providence Health Care Research Ethics Board to use the data for research purposes. Written consent via a standardized consent form approved by the Research Ethics Board verified participant consent prior to any data collection or engagement in the study. Participants who declined to participate, before, during or after data collection or otherwise did not participate remained eligible for treatment and were not disadvantaged in any other way by not participating in the study.

### HIV/AIDS Drug Treatment Program

This study was conducted at the Drug Treatment Program (DTP), at the BC Centre for Excellence in HIV/AIDS, which is mandated by the provincial government to distribute antiretroviral medications free of charge to all eligible HIV-positive individuals. The DTP distributes medications in accordance with the BC Therapeutic Guideline Committee, which have remained consistent with those from the International AIDS Society-USA between 1996 and the most recent revision in 2010 [19]. Details regarding the distribution of antiretroviral medications though the DTP have been previously described in depth [20]. In brief, physicians enter individuals into the DTP when they are first prescribed ART. The enrolling physician must complete a drug request form, which acts as a legal prescription and is used to compile baseline information including past HIV-specific drug history, CD4 cell counts, plasma HIV RNA levels, current drug requests, and enrolling-physician data.

### Study Subjects and Data Collection

Between August 1, 1996 and June 30, 2002, a total of 1,011 participants completed enrollment surveys, which elicited socio-demographic, clinical and behavioral data including the 20-item CES-D scale. Four hundred and forty-eight (44%) were excluded because they did not complete all 20 questions on the CES-D scale, thus 563 were included in this analysis. A comparison of the included and excluded participants showed that, while there is no significant age difference, the excluded group had more females, higher median CD4 cell counts, lower viral load baseline measurements, and fewer individuals that had completed high school. Participants were ≥18 years of age and naïve to antiretroviral therapy when they started HAART.

### CES-D 20 and CES-D 10

The CES-D 20 component of the survey [3] was administered and completed by HIV-positive participants at their enrollment in the provincial Drug Treatment Program. In brief, subjects responded to each item of the scale by rating the frequency of each mood or symptom “during the past week” on a four-point scale. A score is assigned by totaling all items (after reversing the positive mood items]. The possible range for the 20-item scale is 0 to 60, and a cut off score of 16 or higher indicates the presence of significant depressive symptoms.

Our shortened scale was selected from a previously validated ten items in an older adult population [6]. A list of all CES-D 20 items is presented in Table 1, with an asterisk identifying the ten items of our proposed shortened scale. In accordance with Andresen, the possible range of the 10-item scale is 0 to 30, and a cut off score of ten or higher indicates the presence of significant depressive symptoms.

**Table 1:** Center for Epidemiologic Studies Depression Scale (CES-D).

1) I was bothered by things that usually don’t bother me.*
2) I did not feel like eating; my appetite was poor.
3) I felt I could not shake off the blues even with the help from my family and friends.
4) I felt that I was as good as other people.
5) I had trouble keeping my mind on what I was doing.*
6) I felt depressed.*
7) I felt that everything I did was an effort.*
8) I felt hopeful about the future.*
9) I thought my life had been a failure.
10) I felt fearful.*
11) My sleep was restless.*
12) I was happy.*
13) I talked less than usual.
14) I felt lonely.*
15) People were unfriendly.
16) I enjoyed life.
17) I had crying spells
18) I felt sad.
19) I felt that people dislike me.
20) I could not get “going”.*

* Indicates items on our proposed CES-D-10.

### Data Analysis

For each individual we compared their responses to the 20-item scale to our proposed 10-items. The agreement between the CES-D 20 and CES-D 10 was measured using the spearman correlation coefficient and the kappa statistic with two-tailed probability for statistical significance. Cronbach’s alpha [21] was used to estimate the internal consistency of the items within the scale. A reliability coefficient exceeding 0.70 was considered acceptable. Sensitivity and specificity were calculated for the proposed CES-D 10, with sensitivity being the probability of identifying correctly those participants with significant depressive symptoms, while specificity measured the probability of identifying correctly those participants without significant depressive symptoms. Factor analysis with varimax rotation was conducted to examine the latent structure of the new scale. All analyses were conducted using SAS version 9.1.3 (SAS, Cary, North Carolina, United States) with a level of significance set at 0.05.

## Results

The baseline characteristics of the 563 participants have been previously reported [1]. Within the sample, the median age was 38 years (IQR: 34–46 years) and 14% reported aboriginal ancestry, as noted in table 2. In addition, 35% of participants had an average annual income of less than $10,000, 29% did not complete high school, 28% had a history of injection drug use, 20% had an AIDS diagnosis at baseline and 23% reported suboptimal adherence (≥95%) in the first year of follow-up (table 2). Clinical characteristics included a median CD4 cell count of 230 cells/µL (IQR: 80-390 cells/ul), and median HIV RNA plasma viral load (pVL) of 120,000 copies/ml (IQR: 43800-280000 copies/ml) at their initiation of antiretroviral therapy.

**Table 2:** Characteristics of sample and characteristics of participants classified by CES-D-10.

	Baseline characteristics,ALL (n = 563)	CES-D 10 (<10)	CES-D (> = 10)	p-value
Age, median (Q1–Q3)	**38(34–46)**	*39(34–46)*	*38(34–45)*	*0.207*
Gender (male), n%	**91%**	*95%*	*87%*	*0.001*
CD4 cell count, (cells/ul), *median(Q1–Q3*	**230 (80–390)**	*230 (110–400)*	*230 (75–380)*	*0.450*
Plasma Viral Load (copies/ml), *median(Q1–Q3)*	**120,000 (43,800–280,000)**	*100,010 (42,000–251,000)*	*131,000 (46,500–324,000)*	*0.036*
Annual income < $10,000, n%	**35%**	*23%*	*46%*	*<0.001*
Do not complete high school, n%	**29%**	*24%*	*37%*	*<0.001*
History of injection drug use, n%	**28%**	*20%*	*37%*	*<0.001*
Aboriginal, n%	**14%**	*14%*	*15$*	*0.969*
AIDS diagnoses, n%	**20%**	*20%*	*20%*	*0.918*
<95% adherence, n%	**23%**	*18%*	*28%*	*0.007*

We initially compared participants categorized as significant vs non-significant depressive scores on the two scales (table 2). The Kappa statistic for the two methods was 0.82 (table 3). In detail, 289 individuals (51%) were classified as having significant depressive symptoms (CES-D-20≥16) and 284 individuals (50%) were classified as having significant depressive symptoms (CES-D-10≥10) (see table 3). These data suggest that the brief scale is able to identify individuals with significant depression with good precision.

**Table 3:** Agreement between CES-D-10 and CES-D-20.

	<16	≥16 (significant depressive symptom)
**<10**	252	27
≥10 (significant depressive symptom)	22	262

* CES-D-10: the 10 item Center for Epidemiologic Studies Depression Scale as defined in table 1.

* CES-D-20: the 20 item Center for Epidemiologic Studies Depression Scale as shown in table 1.

Kappa Statistic (with 95% CI)  = 0.82 (0.77–0.87).

Sensitivity (with 95% CI)  = 91%(87%–94%).

Specificity (with 95% CI)  = 92%(88%–95%).

Positive predictive value (with 95% CI)  = 92%(89%–95%).

Negative predictive value (with 95% CI)  = 90%(86%–94%).

A factor analysis was conducted to reveal the internal structure of the two scales (as seen in Table 4). Factor 1 was made up of eight items that are all negative statements on patient’s daily life and feeling (all with correlation >0.5) and the remaining two items that are both positive statements were loaded heavily on factor 2 (both with correlation >0.7). The structure clearly revealed two independent factors of the CES-D 10, which were similar to the “negative (depressed) affect” and “positive” affect of the original version of the CES-D. The two factors together explained nearly 60% of the variation of the data.

**Table 4:** Factor Structure of CES-D-10.

	Factor Loading	
	Factor 1	Factor 2
**Factor 1:**48% variationEigenvalue = 3.74		
Everything was an effort	0.77	−0.14
Could not get going	0.74	−0.20
Had trouble keeping mind	0.73	−0.17
Bothered by things	0.69	−0.19
Felt depressed	0.66	−0.50
Sleep was restless	0.65	−0.16
Felt lonely	0.56	−0.45
Felt fearful	0.55	−0.44
**Factor 2:**10% variationEigenvalue = 2.15		
Felt hopeful	−0.05	0.87
Was happy	−0.30	0.77

* CES-D-10: the 10 item Center for Epidemiologic Studies Depression Scale as defined in table 1.

## Discussion

In our analysis, the CES-D-10 scale accurately predicts significant depressive symptoms with good precision in comparison to the 20-item scale. While the reliability of the shortened scale in screening for depressive symptoms in healthy older adults has been demonstrated [6], our findings extend previous reports of the reliability of shortened scales to HIV+ populations who are treatment-naïve at time of enrollment. The factor analysis concluded that the factors most associated with predicating outcomes in the 20-item scale were paralleled in the 10-item scale. The current study provides evidence that the 10-items selected here from the original CES-D scale can be a reliable and a valid measure of significant depressive symptoms among HIV-positive participants.

Given the length of comprehensive questionnaires used to survey the well-being and health of HIV-positive research participants, a shortened scale is needed to allow for a more rapid assessment of significant depressive symptomology, while still maintaining a high degree of sensitivity. This shortened tool will prove to be especially relevant when surveying populations faced with chaotic and un-structured daily lives that may find a 20-item scale too onerous. A number of studies demonstrate a high prevalence of depression among HIV-positive populations and the negative health outcomes of this co-morbidity, which again underlines the importance of this shortened scale [12]. This tool will improve the ability of clinical and public health researchers to assess depressive symptoms among HIV-positive individuals.

### Limitations

There are several limitations of this study. Future studies should examine the test-retest reliability of the CES-D-10 item scale. While we have shown the reliability of this scale using internal consistency, measured by Cronbach’s alpha [21], unfortunately, unlike previous studies [6], [7], where these authors assessed the test-retest reliability, we were unable to assess the test-retest reliability because these participants were not followed up with the same instrument items. However, previous research has used the same ten items to demonstrate that the test-retest reliability of the scale has been satisfied [6].

Our study did not compare the CES-D 10 with clinical diagnoses of depression made by licensed psychiatrists, as the BC-CfE does not collect or link to this data. Although the original CES-D was not designed as a clinical diagnostic tool, the shorter instrument may still benefit from comparisons with clinical criteria of depression, such as the Research Diagnostic Criteria (RDC) or the Diagnostic Interview Survey (DIS).

With a potential sample size of 1,011 for this analysis, nearly half (44%) were excluded because they failed to complete the entire 20-question CES-D scale. Participants that did not complete the entire scale were more likely to be female, have a higher median CD4 cell count, lower viral load baseline measurements and to have not completed high school. Factors such as gender, viral load and education are also found to differ significantly between those characterized by depressive symptoms in our study. The authors hypothesize that individuals may have felt these questions were irrelevant as their physical health is considered to be satisfactory. Another reasoning for this trend is that these are populations that are socially marginalized and have competing priorities that don’t allow for mental health to be a concern, suggesting that a large proportion of the population is being overlooked in terms of their potential depressive symptoms.

### Conclusion

Our analysis of the shortened 10-item CES-D scale attained satisfactory prediction accuracy and reliability in assessing significant depressive symptoms among HIV-positive patients. The validation of this shortened scale provides a useful tool for researching depressive symptomology among HIV-positive populations. We further recommend that our 10-item scale be validated among other populations.
